# Transparent Electrode Based on Silver Nanowires and Polyimide for Film Heater and Flexible Solar Cell

**DOI:** 10.3390/ma10121362

**Published:** 2017-11-29

**Authors:** Xin He, Feng Duan, Junyan Liu, Qiuming Lan, Jianhao Wu, Chengyan Yang, Weijia Yang, Qingguang Zeng, Huafang Wang

**Affiliations:** 1 School of Applied Physics and Materials, Wuyi University, Jiangmen 529020, Guangdong, China; duanfeng0922@163.com (F.D.); liujunyanwyu@126.com (J.L.); wyuqiuminglan@yeah.net (Q.L.); jhwuwyu@126.com (J.W.); yang_cy18phy@126.com (C.Y.); yangweijia5377@126.com (W.Y.); 2 School of Information Engineering, Wuyi University, Jiangmen 529020, Guangdong, China; 3 School of Mechanical Engineering and Automation, Wuhan Textile University, Wuhan 430200, Hubei, China; wanghfhust@163.com

**Keywords:** transparent electrode, silver nanowires, polyimide, film heater, solar cell

## Abstract

Transparent, conductive, and flexible Ag nanowire (NW)-polyimide (PI) composite films were fabricated by a facile solution method. Well-dispersed Ag NWs result in percolation networks on the PI supporting layer. A series of films with transmittance values of 53–80% and sheet resistances of 2.8–16.5 Ω/sq were investigated. To further verify the practicability of the Ag NWs-PI film in optoelectronic devices, we utilized it in a film heater and a flexible solar cell. The film heater was able to generate a temperature of 58 °C at a driving voltage of 3.5 V within 20 s, indicating its potential application in heating devices that require low power consumption and fast response. The flexible solar cell based on the composite film with a transmittance value of 71% presented a power conversion efficiency of 3.53%. These successful applications proved that the fabricated Ag NWs-PI composite film is a good candidate for application in flexible optoelectronic devices.

## 1. Introduction

Over the last several decades, conductive metal-oxide films represented by indium tin oxide (ITO) have been widely used as transparent electrodes in optoelectronic devices [1,2,3,4]. While these flexible devices have attracted increasing attention in recent years, there are still insurmountable difficulties faced by ITO in terms of its further development. Although ITO has excellent optoelectronic properties, the scarcity of indium, the expensive vacuum deposition process and its fragile ceramic nature make it inappropriate for use in flexible or stretchable devices [5,6,7]. Therefore, several materials have been utilized to prepare flexible and transparent electrodes for the replacement of ITO, including conducting polymers [8,9,10], carbon nanotubes [11], graphene [12,13], and metal nanowires [14,15,16,17,18,19,20,21]. Among these substitutable materials, Ag NWs have found a great number of applications as the transparent electrode due to their excellent optoelectronic performance, compatibility with flexible substrates, and easy adaptation for large-scale fabrication [14,16,18,19,20,22]. However, different optoelectronic devices require transparent electrodes with different performances. For example, the organic light-emitting diode (OLED) and solar cell need electrodes with low surface roughness to avoid short circuit between the electrode and the active layer [23,24,25]. Film heaters demand a transparent electrode with good adhesion to the substrate, and excellent conductivity and stability, resulting in a device suitable for long-term use [5,26,27,28].

Therefore, researchers have developed several techniques for improving the performance of Ag NW electrodes in order to accommodate multiple applications. For example, Ag NWs were coated with PEDOT:PSS to decrease the surface roughness and increase the conductivity of the transparent electrode for applications in solar cells and touch panels [29,30,31]. Moon et al. reported that the Ag NWs formed a random percolating network embedded between ZnO layers, resulting in excellent thermal stability, adhesiveness, and flexibility, as well as high electrical conductivity [32]. Furthermore, the Ag NW networks were usually combined with polymers (polyvinyl alcohol, polymethacrylate, polyimide (PI), polydimethylsiloxane) to achieve flexibility, stretchability, and good adhesion to the substrates [18,33,34,35,36,37]. In this work, we fabricated Ag NW-PI composite film with good conductivity, high-temperature tolerance, and transparency. We improved the fabrication technology of the composite film to obtain a uniform NW distribution across the PI supporting layer. The Ag NW networks were partially embedded into the PI layer by transfer technology, which was effectively able to improve the adhesion of the conducting networks and reduce the surface roughness of the resulting film.

Additionally, the optoelectronic performance of the composite films was able to be varied by adjusting the density of the Ag NW networks. Thus, we were able to choose the conducting films with different properties (optoelectronic properties, physical surface properties, chemical and thermal stability) as the transparent electrode in order to accommodate the various requirements of optoelectronic devices. The Ag NW-PI composite films were successfully applied in the film heaters and solar cells in this study. By controlling the optoelectronic properties of the composite film, various levels of heating performance could be achieved for the film heaters, and various current density-voltage characteristics could be obtained for the solar cells. These investigations confirm that the Ag NW-PI composite film is an excellent candidate for meeting diverse application requirements for flexible and transparent electrodes.

## 2. Experimental Section

### 2.1. Fabrication of Transparent Electrode Based on Silver Nanowires and Polyimide

**Figure 1:**
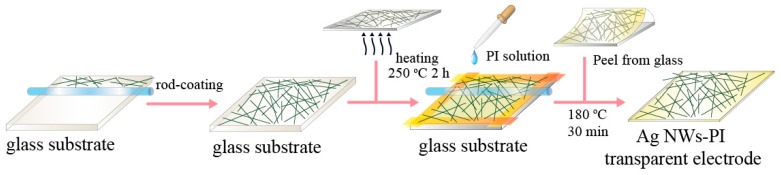
Schematic illustration of the fabrication process of Ag NW-PI (Ag nanowire-polyimide) transparent conductive film.

High-yielded Ag NWs were synthesized using the modified hydrothermal method reported by our group in [35]. 0.75 mmol of silver nitrate and 1 mmol of glucose were dissolved in a beaker at room temperature. Then, 0.15 mmol ferric sulfate was added and magnetically stirred for several minutes. Following this, 2.25 g poly (vinyl pyrrolidone) (K30) (PVP) was introduced into the solution. The resultant solution, with a volume of 40 mL, was transferred to a Teflon autoclave of 100 mL capacity, which was sealed and heated at 180 °C for 6 h. The as-obtained precipitate was centrifuged and dispersed in ethanol to obtain Ag NW dispersion. Figure 1 displays a schematic illustration of fabrication process of the Ag NWs-PI composite film, the detailed procedure is described as follows. A uniform Ag NW film was firstly coated on a cleaned bare glass substrate by pulling a glass rod over the Ag NW ethanol dispersion. The soda-lime glass substrate was cleaned by sonication in acetone, de-ionized water and ethanol for 10 min each, after which they were dried under infrared light. Although PVP is a necessary reactant to form the one-dimensional nanostructure by controlling the growth rates of various crystal faces of silver, the PVP on the NW surface should be removed after the reaction due to its poor conductivity. Thus, the Ag NW film on glass substrate was heated at a constant temperature of 250 °C for 2 h (the oven was preheated to 250 °C) in order to remove surfactant PVP on the NW surface and melt the junctions of the NWs [38]. After heat treatment, the disconnected NWs could be melted together to form a connected network, and the PVP on the surface of the NWs could be combusted, leading to a decrease in the sheet resistance from the initial thousands of Ohms per square to several Ohms per square. After that, four sides of the Ag NW film were affixed using transparent tape to control the thickness of PI layer. Following that, 18 wt % PI in dimethylformamide solution was dropped onto the Ag NW film with the assistance of a glass rod to flatten the PI layer, and then it was cured at 180 °C for 30 min at a heating rate of 1 °C/min (the oven was heated from room temperature to 180 °C within 150 min, and then maintained at 180 °C for 30 min). Finally, the upper polymer film was peeled off from the glass substrate and the conducting network of the Ag NW was transferred onto the surface of the PI layer. The cured PI layer was able to support the conducting network, and the Ag NWs could be partially embedded into the cured PI layer, resulting in the Ag NW-PI composite film. The thickness of the composite film was approximately 110–140 μm.

### 2.2. Fabrication of Film Heater and Flexible Solar Cell

The film heater based on the Ag NWs-PI transparent electrode is produced by applying a direct current (DC) voltage through copper conducting tape at the two opposite edges of the film. The area of the composite film was 4 cm × 4 cm. The width of the copper conducting tape was 0.5 cm, so the distance between the two tapes was 3 cm. The temperatures generated by the film heaters were measured using an IR thermometer.

In the flexible solar cells, poly[(9,9-bis(3′-(*N*,*N*-dimethylamino)propyl)-2,7-fluorene)-alt-2,7-(9,9-dioctylfluorene)] (PFN) was used as an interfacial modification layer to help collect and extract the charge carriers. The PFN layer, with a thickness of 10 nm, was spin-coated onto the surface of the Ag NW-PI electrode, and then poly[[4,8-bis[(2-ethylhexyl)oxy] benzo[1,2-b:4,5-b′] dithiophene-2,6-diyl] [3-fluoro-2-[(2-ethylhexy)carbonyl]thieno[3,4-b]thiophenediyl]] (PTB7): [6,6]-phenyl C_71_ butyric acid methyl ester (PC_71_BM) blend solution with a concentration of 25 mg/mL was spin-coated onto the top of the PFN layer, resulting in a PTB7:PC_71_BM layer with a thickness of 95 nm. Finally, MoO_3_ with a thickness of 10 nm and an Ag layer with a thickness of 100 nm were deposited onto the active layers by thermal evaporation. The resulting device structure was Ag NW-PI electrode/PFN/PTB7:PC_71_BM/MoO_3_/Ag. The effective area was 14 mm^2^, and was determined by using a patterned metal mask.

### 2.3. Characterization

The morphologies of the films were observed using a field emission scanning electron microscope (FESEM) (NoVaTM Nano SEM 430, FEI, Hillsboro, OR, USA). A transmission electron microscope (TEM) measurement was carried out with a JEOL-2100F electron microscope (JEOL, Tokyo, Japan) to investigate the diameter of the nanowires. The transmittances of the films were recorded using a UV-Vis spectrophotometer (UV2550, Shimadzu, Kyoto, Japan) with a xenon lamp as light source, and the reference spectra were evaluated from air. The sheet resistances of the films were characterized using a four-point probe system (SZ-82, Suzhou Telecommunication, Suzhou China), and the final values were averaged from nine representative points on the conductive surfaces. The heating performances of the films were measured using a two-terminal side contact configuration. The applied DC voltage was supplied by a power supply (ITECH, IT6720, San Luis Obispo, CA, USA) to the heater through two pieces of copper conductive tape pasted at the film edges. The temperatures and heat distributions of the transparent films were evaluated using an IR thermal imager (Fluke Ti32, Washington, DC, USA). The current density versus voltage (*J-V*) characteristics of the fabricated solar cells were recorded by a Keithley 2400 source meters under illumination of an AM 1.5G solar simulators with an intensity of 100 mW/cm^2^ (Sun 2000 Solar Simulator, Abet Technologies, Inc., Milford, CT, USA). External quantum efficiency (EQE) measurements in the wavelength range of 300–800 nm were performed on an Enlitech QE-R spectral response measurement system (Enli Tech Ltd., Kaohsiung, Taiwan).

## 3. Results and Discussion

Figure 1 shows the schematic illustration of the fabrication process of the Ag NW-PI composite film. The Ag NW dispersion was coated on the glass substrate to result in the crossed networks. Uniform conducting networks with various densities were obtained by pulling the glass rod layer by layer in mutually perpendicular directions. It is worth noting that the Ag NW ethanol dispersion used to fabricate the Ag NW film on the glass substrate had a constant concentration. The difference in the transmittance of the films is realized by changing the number of coating layers and pulling speed. Additionally, some ethanol dispersion was drawn out of the substrate during the coating process, resulting in a loss of Ag NWs. Thus, we distinguished the densities of the Ag NWs in the films by the transmittances. As the transmittance of the film decreased, the density of the Ag NWs increased. Then, the fabricated Ag NW film was heated to improve the film conductivity. Following that, the PI layer was coated onto the surface of Ag NW film by thermal curing. The PI film embedded with the Ag NW networks could be peeled off from the substrate to obtain the Ag NW-PI transparent electrode. We measured the transmittances of the composite film before and after peeling by 3M Scotch tape. The transmittance of the film at 550 nm showed less than 1% deviation after peeling, indicating good adhesion of the conductive networks to the PI layer due to the embedding of the Ag NWs into the PI supporting layer during the transfer process.

**Figure 2:**
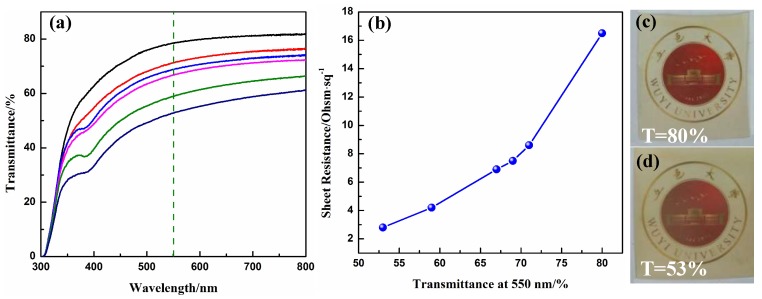
(**a**) Optical transmittance spectra of the Ag NWs-PI transparent electrodes with various transmittances of 53%, 59%, 67%, 69%, 71% and 80% at 550 nm; (**b**) Plots of sheet resistance versus transmittance at 550 nm of the Ag NWs-PI transparent electrodes (blue ball represents the value of sheet resistance at a certain transmittance at 550 nm); (**c**,**d**) Photographs of the composite films with the transmittances of 80% and 53%, respectively, overlaid on the logo of Wuyi University.

Figure 2a presents the optical transmittance spectra of the Ag NWs-PI composite films with various densities of the Ag NWs by taking air as the reference. The transmittance of the films at 550 nm was 53%, 59%, 67%, 69%, 71% and 80%, while the corresponding sheet resistance was 2.8, 4.2, 6.9, 7.5, 8.6 and 16.5 Ω/sq, respectively. The sheet resistances were averaged from nine representative points on the conductive surfaces. We calculated the standard deviation (SD) of the sheet resistance for the conductive films using Formula (1). The values of SD for the films with transmittance of 80%, 71%, 69%, 67%, 59% and 53% were, respectively, 9.3, 6.2, 3.0, 2.8 1.6 and 1.5.

(1) $$SD=\sqrt{\frac{\sum_{i=1}^{n}\left(x_{i}-\bar{x}\right)}{n-1}}$$

The variation of sheet resistance with transmittance is depicted in Figure 2b, illustrating that the conductivity of the composite films is improved as the transmittance decreased. Higher Ag NW density provides more conductive pathways for the electrons, and less free space in between the NWs in the films, leading to the high conductivity and low transmittance of the film.

Figure 2c,d shows photographs of the Ag NWs-PI composite films with transmittances of 80% and 53% at 550 nm, indicating that the conducting networks were uniformly dispersed across the polymer layer. It is sufficiently transparent that the logo of Wuyi University below the films can be clearly identified, even for the film with a transmittance of 53%. However, the composite films exhibited a light-yellow color due to the thick PI layer. The transparency can be further increased by reducing the thickness of the polymer layer, as shown in Figure S1.

**Figure 3:**
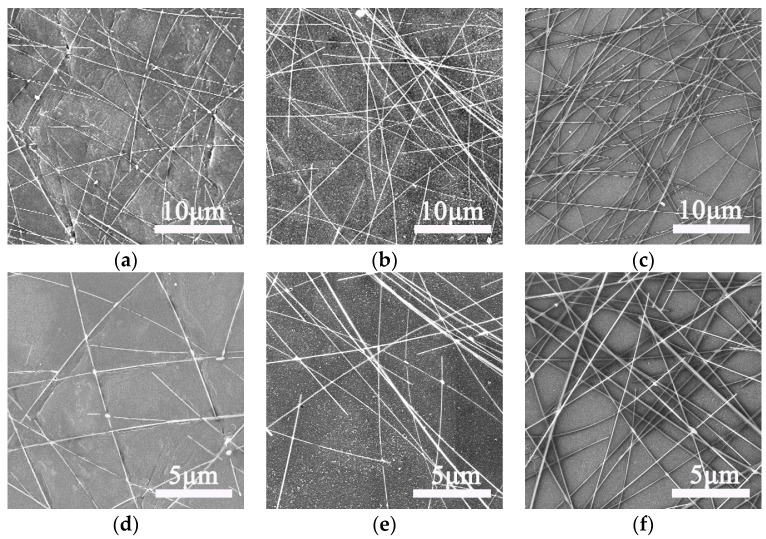
Low- and high-magnification SEM (scanning electron microscope) images for the Ag NW-PI transparent electrodes with transmittances of 71% (**a**,**d**), 67% (**b**,**e**) and 53% (**c**,**f**), respectively.

Figure 3a–f present low- and high-magnification SEM images for the Ag NW-PI films with transmittances of 71%, 67% and 53%, respectively. The NWs display average diameters of approximately 50 nm, which is confirmed by the typical TEM image with a large magnification, as shown in Figure S2. The length of the NWs reached dozens of micrometers, and they were intersecting each other to form random networks across the substrate surface. Additionally, the SEM image of the film with a transmittance of 80% is shown in Figure S3. It can be seen that, as the transmittance of the composite films decreased, the free spaces among the NWs were also reduced, and the density of the NWs gradually increased. Therefore, the connections of the NWs were improved, providing more electron pathways in the percolation networks, which is consistent with the optoelectronic properties of the composite films. We also tested the flexibility and mechanical stability of the as-fabricated film. The resistance of the film with the initial value of 6.7 Ω/sq after 300 bending cycles was measured, and the variation in resistance versus cycle numbers is shown in Figure S4. The result reveals that the resistance of the film showed less than 3% deviation after 300 bending cycles, indicating the film presents a high tolerance to bending and good flexibility.

**Figure 4:**
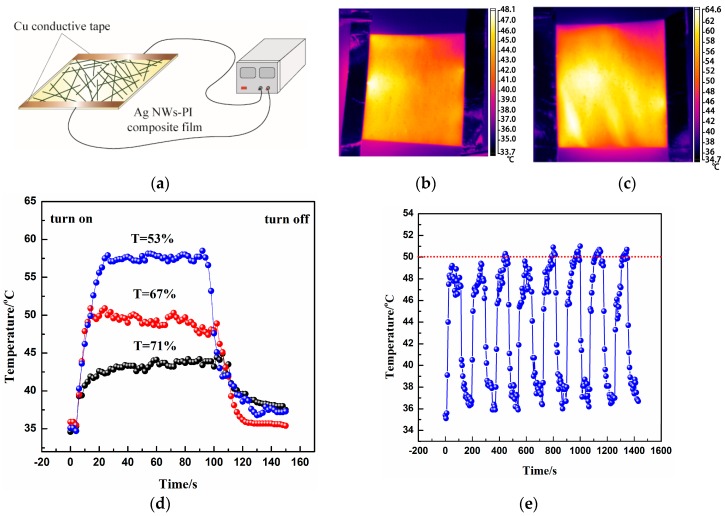
(**a**) Schematic illustration of a film heater based on the Ag NW-PI film; Infrared thermal images of the composite films with the transmittance of 71% (**b**) and 53% (**c**) at 3.5 V; (**d**) Evolution of the temperature of Ag NWs-PI composite films with the transmittance of 71%, 67% and 53% at 3.5 V; (**e**) cycling performance of the film heater with transmittance of 67% at 3.5 V.

Figure 4a is a schematic illustration of the film heater based on the Ag NWs-PI electrode. By applying a given DC voltage to the copper conductive tape, the film can generate Joule heat. The temperature of the film surface was recorded with an IR thermometer. Figure 4b,c shows typical infrared thermal images of the Ag NWs-PI films with transmittances of 71% and 53% at a driving voltage of 3.5 V, respectively, demonstrating the heat-distributions of the films. Figure 4d presents a temperature evolution of the Ag NW-PI composite films with transmittances of 71%, 67% and 53% at 3.5 V. The heating and cooling times were 100 s and 50 s, respectively. The film cooled naturally to room temperature without any cooling method. The temperatures of the films with various transmittances could be rapidly increased from room temperature to saturation value within 20 s, indicating the fast heat-response of the Ag NW-PI film heater. The saturated temperatures of the films with the transmittances of 71%, 67% and 53% were 44, 50 and 58 °C, respectively, and were obtained directly from the IR thermometer. As the transmittance of the films decreased, the temperature generated increased at a given driving voltage, which is attributed to the increase in electron pathways in the conducting networks. Furthermore, repeated heating tests were conducted by turning on and off the voltage of 3.5 V; the cycling curve of the film heater with a transmittance of 67% is shown in Figure 4e. The saturated temperature of each cycle was around 50 °C, presenting relatively stable heating cycles.

**Figure 5:**
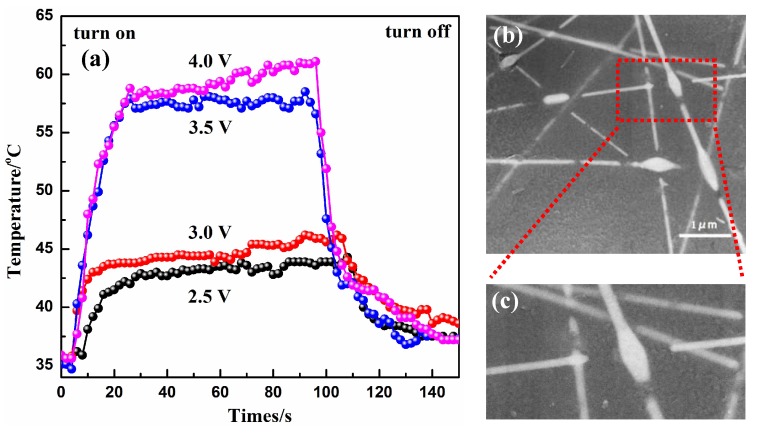
(**a**) Evolution of generated temperature of the Ag NWs-PI film with transmittance of 53% at various voltages from 2.5 to 4 V; (**b**) SEM image of the composite film after heating at a high voltage; (**c**) magnified SEM image of red rectangle area in (**b**).

Figure 5a presents the temperature profiles of the Ag NW-PI composite film with the transmittance of 53%, for driving voltages varying from 2.5 to 4 V. When the potential was set at 2.5, 3.0, 3.5 and 4.0 V, the temperature generated by the film heater was 44, 46, 58 and 61 °C, respectively, demonstrating that the saturation values of the film heaters were elevated when the input voltage was raised. However, when the potential was further increased to 5.0 V, the temperature initially increased, but then decreased after continuously heating for several minutes. In this case, several NWs probably became disconnected, destroying the conductive networks, resulting in a decrease in temperature. The SEM image of the film after heating at 5.0 V for several minutes is depicted in Figure 5b, indicating that several junctions of the NWs had become disconnected. A magnified image of the red rectangle area in Figure 5b is displayed in Figure 5c. It can be seen that the disconnection is only observed at the junctions, not at the straight part of the NWs. There are high resistances at the junctions, resulting in the generation of a large amount of heat under high input voltage in a short time. The NWs at these locations were probably melted, disconnecting the NWs and enlarging the junction size. In this case, the conductive networks were probably destroyed, and the performance of the film heater was decreased. A similar phenomenon has also been reported elsewhere [39].

Therefore, the fabricated film heaters based on the Ag NWs-PI composite films with good optoelectronic properties exhibit flexibility, low-voltage driving, and fast heat-response. A stable temperature could be obtained at a low potential, which is suitable for the device’s required low power consumption and high safety for human beings.

**Figure 6:**
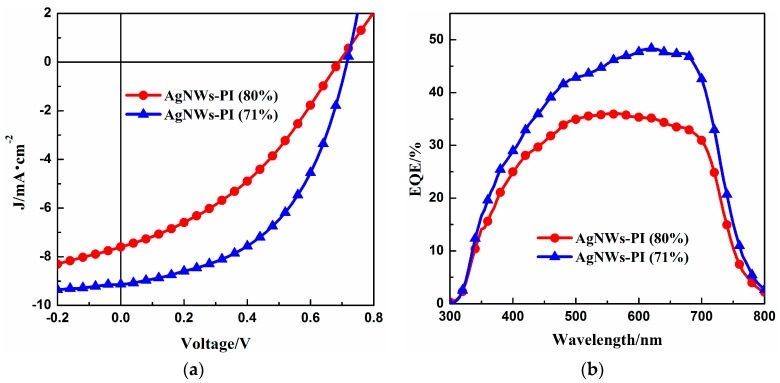
*J-V* characteristics (**a**) and EQE (external quantum efficiency) spectra (**b**) of the solar cells based on the Ag NWs-PI transparent electrodes with transmittances of 80% and 71%, respectively.

In order to evaluate the performance of the Ag NW-PI transparent electrode in the other optoelectronic device, we applied it in a polymer solar cell with the device structure Ag NW-PI electrode/PFN/PTB7:PC_71_BM/MoO_3_/Ag. The photovoltaic performance of the two solar cell devices based on the Ag NW-PI electrodes with transmittances of 80% and 71% under AM 1.5G illumination conditions are represented by the current density-voltage (*J-V*) curves in Figure 6a. The device parameters, including short-circuit density (*J_sc_*), open-circuit voltage (*V_oc_*), fill factor (*FF*, which is defined as the ratio of maximum power to the cell of the open-circuit voltage and short-circuit current), and power conversion efficiency (PCE), were deduced from the *J-V* characteristics. The parameter values are summarized in Table 1.

In comparison, the flexible solar cell based on the Ag NWs-PI electrode with the transmittance of 71% exhibited a higher short-circuit current (*J_sc_*) of 9.95 mA/cm^2^, an open-circuit voltage (*V_oc_*) of 0.72, and a fill factor (*FF*) of 50%, leading to a PCE of 3.53%. Although more light could be transmitted into the absorber layer of the polymer solar cell based on the Ag NW-PI electrode with 80% transmittance, the solar cell device only exhibited a *J_sc_* of 7.15 mA/cm^2^, a *V_oc_* of 0.70, a *FF* of 37%, and a PCE of 1.96%. Additionally, the device based on the film with the transmittance of 80% presented lower shunt resistance and larger leaking current. Because the conductive networks were partially embedded into the PI layer, there was no significant difference in the surface roughness between the films with a transmittance of 80% and of 71%. The difference in the shunt resistance and leaking current was mainly dependent on the conductivity of the films, not the surface roughness. The high conductivity of the transparent electrode could lead to more charge carriers transporting to the electrode surface, which would decrease the leaking current and increase the shunt resistance.

Figure 6b shows EQE spectra of the solar cell devices based on the Ag NW-PI electrodes with transmittances of 80% and 71%. Both of the devices had relatively flat EQE curves at wavelengths of 450–700 nm, compared to those in the ranges of 300–450 nm and 700–800 nm. The maximum value of EQE was 36% and 48% for the solar cells with the Ag NW-PI electrodes with transmittances of 80% and 71%, respectively, revealing an improvement in device performance when the transparent electrode with a transmittance of 71% was used. The EQE comparison also indicates that a higher conductivity plays a positive role in generating transporting and/or collecting charge carriers, which agrees well with the results for *J-V* characteristics in Figure 6a.

## 4. Conclusions

In summary, we demonstrated the fabrication of the Ag NW-PI composite film. The transparent conductive films were successfully utilized in film heaters and flexible solar cells. On the one hand, the effects of Ag NW density and input voltage on the heating performance of the film heater were investigated. The heater exhibited a rapid thermal response, low driving potential, and stable cyclic use. On the other hand, a flexible solar cell based the composite film with 71% transmittance exhibited *J_sc_* of 9.95 mA/cm^2^, *V_oc_* of 0.72 V, *FF* of 50%, PCE of 3.53% and EQE of 48%. These investigations demonstrate that the performances of both the film heater and the solar cell were increased with the conductivity improvement of the composite films, which is ascribed to the increase of electron pathways in the networks. Therefore, it is believed that the Ag NW-PI composite films would be a promising candidate for next-generation devices.

## Figures and Tables

**Table 1:** Photovoltaic characteristics of the solar cells based on the Ag NW-PI (Ag nanowire-polyimide) transparent electrodes with transmittances of 80% and 71% under AM 1.5G illumination.

Device	*J_sc_* (mA·cm^−2^)	*V_oc_* (V)	*FF* (%)	PCE (%)	EQE (%)
Ag NWs-PI electrode(80%)/PFN/PTB7:PC_71_BM/MoO_3_/Ag	7.15	0.70	37	1.96	36
Ag NWs-PI electrode(71%)/PFN/PTB7:PC_71_BM/MoO_3_/Ag	9.95	0.72	50	3.53	48

## Supplementary Materials

The following are available online at www.mdpi.com/1996-1944/10/12/1362/s1. Figure S1: Plots of thickness of PI film versus transmittance at 550 nm, Figure S2: TEM image with a large magnification of the Ag NWs, Figure S3: SEM image of the composite film with the transmittance of 80%, Figure S4: Variations in resistance of Ag NWs-PI composite film versus bending cycles.
