# Supplementary material for: Transparent Electrode Based on Silver Nanowires and Polyimide for Film Heater and Flexible Solar Cell
Source: Materials (Basel). 2017 Nov 29;10(12):1362. doi: 10.3390/ma10121362 (PMC5744297; doi:10.3390/ma10121362)
Supplement: Supplementary file 1 [file materials-10-01362-s001.pdf]

materials

*Article*

# Transparent Electrode Based on Silver Nanowires and Polyimide for Film Heater and Flexible Solar Cell

**Xin He [1,*], Feng Duan [1,2], Junyan Liu [1], Qiuming Lan [1], Jianhao Wu [1], Chengyan Yang [1], Weijia Yang [1], Qingguang Zeng [1,*] and Huafang Wang [3]**

[1] School of Applied Physics and Materials, Wuyi University, Jiangmen 529020, Guangdong, China; duanfeng0922@163.com (F.D.); liujunyanwyu@126.com (J.L.); wyuqiuminglan@yeah.net (Q.L.); jhwuwyu@126.com (J.W.); yang_cy18phy@126.com (C.Y.); yangweijia5377@126.com (W.Y.)

[2] School of Information Engineering, Wuyi University, Jiangmen 529020, Guangdong, China

[3] School of Mechanical Engineering and Automation, Wuhan Textile University, Wuhan 430200, Hubei, China; wanghfhust@163.com

* Correspondence: hexin@mail.wyu.edu.cn (X.H.); zengqg1979@126.com (Q.Z.)

Received: 27 September 2017; Accepted: 23 November 2017; Published: 29 November 2017

**Supplementary**

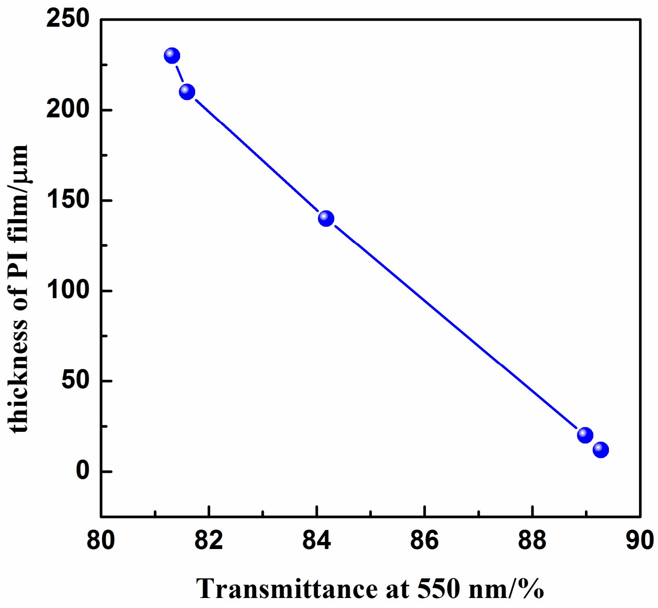


Figure S1. Plots of thickness of PI film versus transmittance at 550 nm

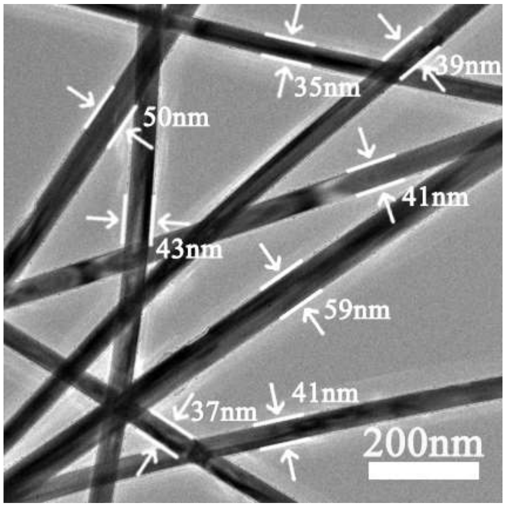


**F**igure S2. TEM image with a large magnification of the Ag NWs

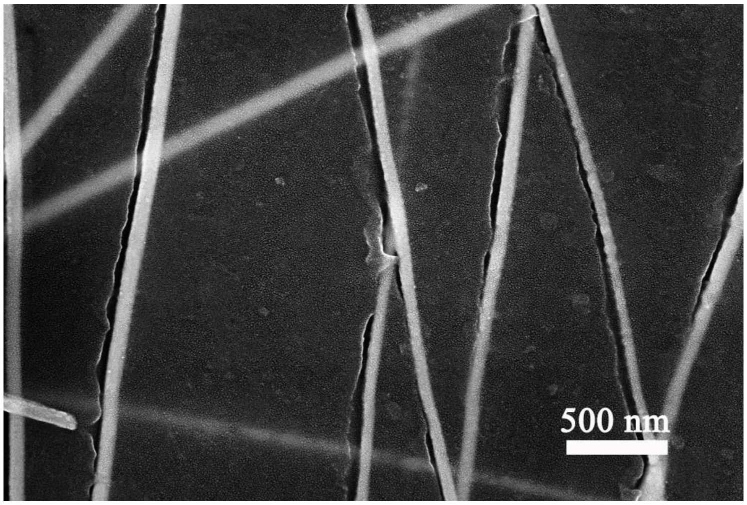


**F**igure S3. SEM image of the composite film with the transmittance of 80%

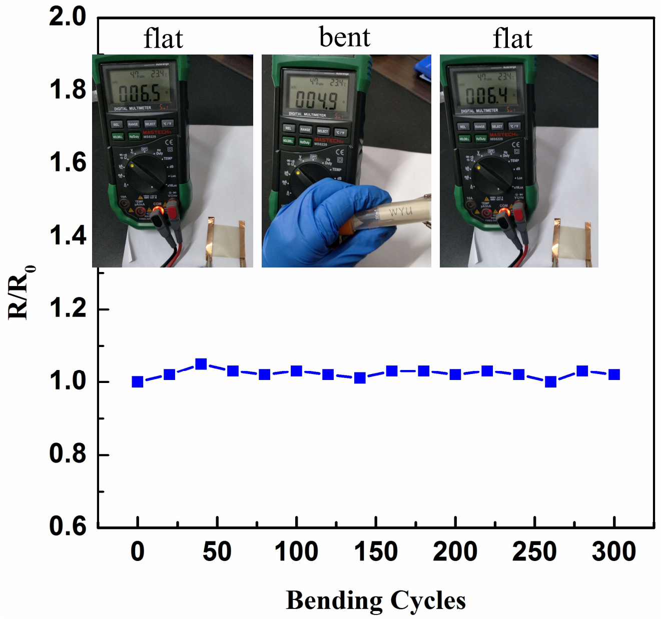


**F**igure S4. Variations in resistance of Ag NWs-PI composite film versus bending cycles

© 2017 by the authors. Submitted for possible open access publication under the terms and conditions of the Creative Commons Attribution (CC-BY) license (http://creativecommons.org/licenses/by/4.0/).
